# *Salvia miltiorrhiza* and Its Compounds as Complementary Therapy for Dyslipidemia: A Meta-Analysis of Clinical Efficacy and In Silico Mechanistic Insights

**DOI:** 10.3390/ph17111426

**Published:** 2024-10-24

**Authors:** Min-Seong Lee, Han-Young Lee, Seung-Hyun Oh, Chang-Bum Kim, Ji-Han Kim, Seung-Hoon Yoo, Yeon-Joo Yoo, Su-Yeon Lee, Byung-Cheol Lee

**Affiliations:** 1 Department of Clinical Korean Medicine, Graduate School, Kyung Hee University, 26, Kyungheedae-ro, Dongdaemun-gu, Seoul 02447, Republic of Korea; min6691@khu.ac.kr (M.-S.L.); hanyoung2357@gmail.com (H.-Y.L.); sshyunbiz@naver.com (S.-H.O.); kinkcb@gmail.com (C.-B.K.); ilpc21@naver.com (J.-H.K.); ysh9306@khu.ac.kr (S.-H.Y.); alice98113@khu.ac.kr (Y.-J.Y.); 2 College of Korean Medicine, Kyung Hee University, Seoul 02447, Republic of Korea; suyeon427@khu.ac.kr

**Keywords:** dyslipidemia, *Salvia miltiorrhiza*, meta-analysis, network pharmacology

## Abstract

**Background/Objectives:** Dyslipidemia is a significant risk factor for atherosclerotic cardiovascular disease (ASCVD), a leading cause of death worldwide. *Salvia miltiorrhiza* Burge is widely used in East Asia for cardiovascular health, showing potential benefits in lowering cholesterol and reducing inflammation. **Methods:** This study systematically reviewed and conducted a meta-analysis of randomized controlled trials (RCTs) to assess the clinical effectiveness of *Salvia miltiorrhiza* in treating dyslipidemia. Moreover, network pharmacology and molecular docking analyses were performed to explore the mechanisms underlying the effects of *Salvia miltiorrhiza*. **Results:** The meta-analysis revealed that when *Salvia miltiorrhiza* is combined with statin therapy, it significantly enhances lipid profiles, including reductions in total cholesterol, low-density lipoprotein cholesterol (LDL-C), and triglycerides and improvements in high-density lipoprotein cholesterol (HDL-C), compared to statin therapy alone. The in silico analyses indicated that *Salvia miltiorrhiza* may influence key biological pathways, such as the PI3K/Akt, JAK/STAT, and HMGCR pathways, which are involved in inflammation, lipid metabolism, and the development of atherosclerosis. **Conclusions:** *Salvia miltiorrhiza* shows potential as a complementary therapy for dyslipidemia, offering additional lipid-lowering and anti-inflammatory benefits.

## 1. Introduction

Dyslipidemia is a key risk factor for atherosclerotic cardiovascular disease (ASCVD), a leading cause of mortality worldwide. Elevated low-density lipoprotein cholesterol (LDL-C) levels in the bloodstream lead to endothelial damage, monocyte recruitment, and plaque formation, culminating in atherosclerotic lesions [1]. Furthermore, oxidized LDL exacerbates this process by impairing nitric oxide production, increasing oxidative stress, and activating nuclear factor kappa B (NF-κB), which promotes inflammation, cell apoptosis, and foam cell development in vascular smooth muscle cells [2]. Thus, the proper management of dyslipidemia, particularly by addressing LDL, is crucial for reducing ASCVD risk [3].

Cholesterol is primarily synthesized in the liver, either through the absorption of lipoproteins from the blood or through de novo synthesis, a process regulated by 3-hydroxy-3-methyl-glutaryl-coenzyme A reductase (HMGCR) [4]. Statins, which inhibit HMGCR, remain the cornerstone of dyslipidemia treatment. However, statin therapy is associated with several side effects, including muscle symptoms such as myalgia and an increased risk of developing diabetes in some patients [5]. Additionally, a significant number of patients on statins do not achieve optimal LDL-C targets, highlighting the need for alternative or additional lipid-lowering therapies [6].

*Salvia miltiorrhiza* Burge, commonly known as Danshen, has been used in traditional East Asian medicine to improve blood circulation and relieve blood stasis [7]. Recent studies have indicated that Tanshinone IIa, one of the major constituents of Danshen, has shown a wide range of beneficial effects, including anti-inflammatory and anti-atherosclerotic properties [8,9]. Although Danshen and its formulations are commonly used in East Asia including Korea and China for treating dyslipidemia and ASCVD, and while some studies have reported favorable effects of Danshen on dyslipidemia, no comprehensive meta-analysis has yet been conducted to evaluate its therapeutic efficacy. Furthermore, the precise mechanisms by which components of Danshen exert their effects on dyslipidemia have been explored but not fully elucidated.

In this study, we conducted a systematic review and meta-analysis of randomized controlled trials (RCTs) to assess the clinical effectiveness of Danshen in the treatment of dyslipidemia. Additionally, we performed mechanism analyses through network pharmacology and molecular docking to investigate the underlying biological pathways involved in Danshen’s action.

## 2. Results

### 2.1. Results of Meta-Analysis

#### 2.1.1. Description of Studies

**Figure 1:**
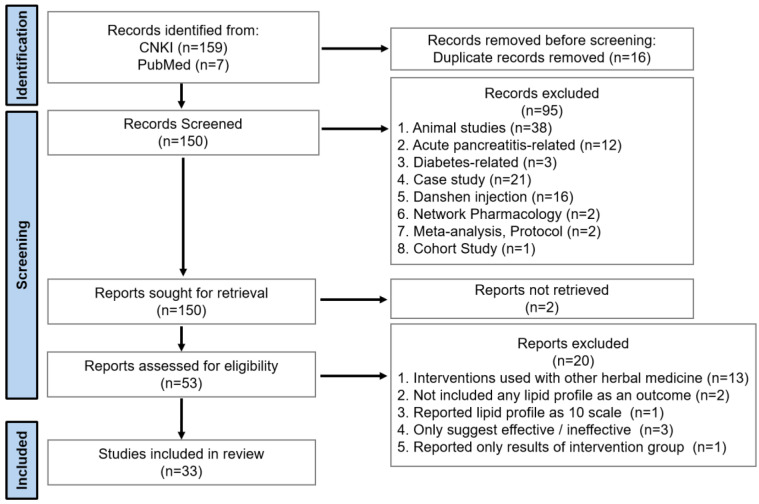
Meta-analysis flow diagram.

A total of 166 relevant studies were identified, with 159 from CNKI and 7 from PubMed. After removing 16 duplicate studies, 150 articles were screened, of which 94 were excluded for the following reasons: animal studies (*n* = 38), acute pancreatitis-related (*n* = 12), diabetes-related (*n* = 3), case studies (*n* = 21), Danshen injection (*n* = 16), network pharmacology (*n* = 2), meta-analysis/protocol (*n* = 2), cohort study (*n* = 1), and reports not retrieved (*n* = 2). The remaining 53 studies were assessed for eligibility (*n* = 56), among which 20 were further excluded for the following reasons: interventions combined with other herbal medicine (*n* = 13), did not include any lipid profiles as an outcome (*n* = 2), reported lipid profile on a 10-point scale (*n* = 1), only suggested effectiveness/ineffectiveness (*n* = 3), and reported only the results of the intervention group (*n* = 1). Finally, 33 studies were included in the analysis. The process is illustrated in Figure 1.

Among the 33 RCTs, 32 were conducted in China and 1 was conducted in the Netherlands. The characteristics of each study are summarized in Table 1. The trials assessed the following four indicators: total cholesterol (TC) (*n* = 33), triglycerides (TGs) (*n* = 29), LDL-C (*n* = 28), and high-density lipoprotein cholesterol (HDL-C) (*n* = 25).

The characteristics of each experiment are summarized in Table 1.

#### 2.1.2. Risk of Bias in the Included Studies

**Figure 2:**
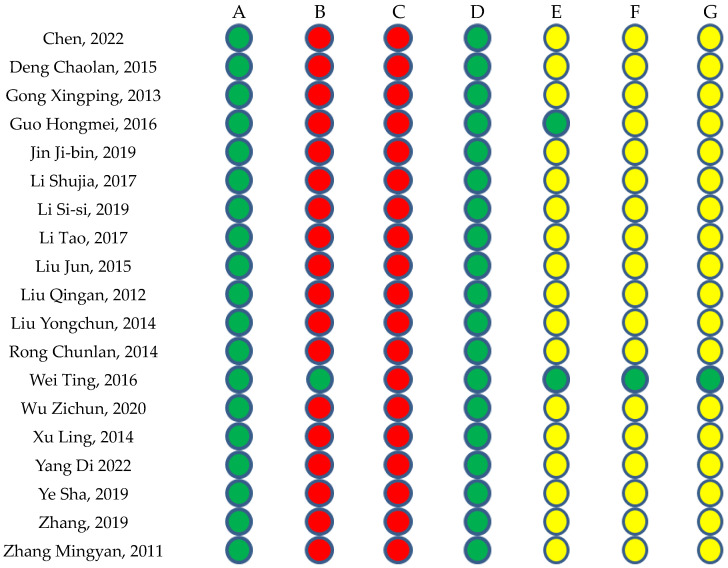

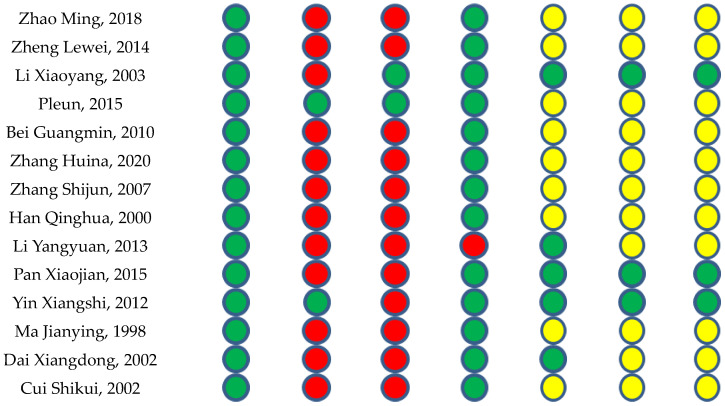
A: Random Sequence Generation (selection bias), B: Allocation Concealment (selection bias), C: Blinding of Participants and Personnel (performance bias), D: Blinding of Outcome Assessment (detection bias), E: Incomplete Outcome Data (attrition bias), F: Selective Reporting (reporting bias), G: Other Bias. Red: high risk, Yellow: uncertain risk, Green: low risk [10,11,12,13,14,15,16,17,18,19,20,21,22,23,24,25,26,27,28,29,30,31,32,33,34,35,36,37,38,39,40,41,42].

A risk of bias table was constructed based on the following criteria: Random Sequence Generation (selection bias), Allocation Concealment (selection bias), the Blinding of Participants and Personnel (performance bias), the Blinding of Outcome Assessment (detection bias), Incomplete Outcome Data (attrition bias), Selective Reporting (reporting bias), and Other Bias. Low risk is indicated in green, uncertain risk in yellow, and high risk in red. The risk of bias assessments for each study are presented in Figure 2 below.

#### 2.1.3. Effective Rate

##### Total Cholesterol

**Figure 3:**
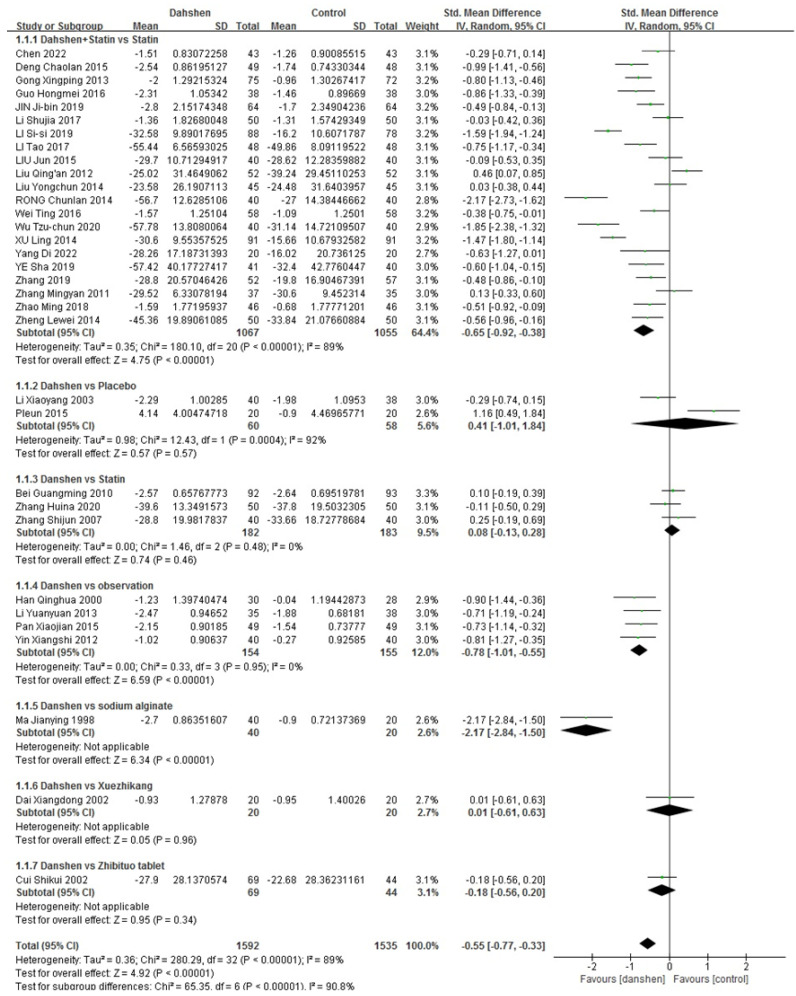
Complete data of total cholesterol [10,11,12,13,14,15,16,17,18,19,20,21,22,23,24,25,26,27,28,29,30,31,32,33,34,35,36,37,38,39,40,41,42].

Twenty-one trials compared the concurrent use of Danshen and statins versus statins alone. Two trials compared Danshen alone versus a placebo. Three trials compared Danshen alone versus statins alone, and four trials compared Danshen alone versus the observation. Additionally, the number of trials comparing Danshen alone versus sodium alginate, Xuezhikang (XZK), or Zhibituo tablets as control groups was one each. The results are presented in Table 2 and Figure 3.

##### Triglycerides

**Figure 4:**
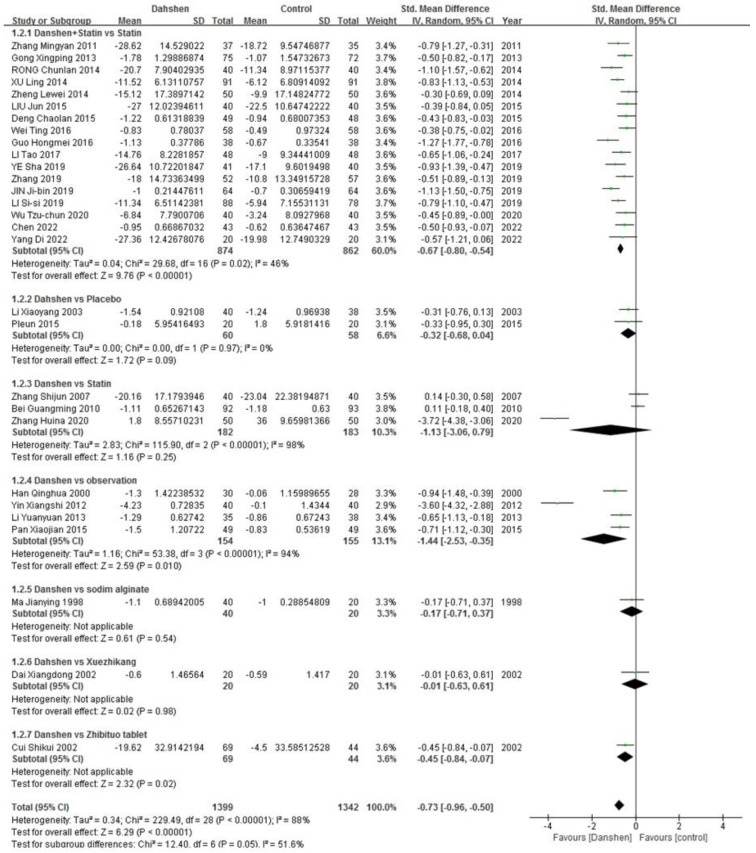
Complete data of triglycerides [10,11,12,13,14,16,17,18,21,22,23,24,25,26,27,28,30,31,32,33,34,35,36,37,38,39,40,41,42].

Seventeen trials compared the concurrent use of Danshen and statins versus statins alone. Two trials compared Danshen alone versus a placebo. Three trials compared Danshen alone versus statins alone, and four trials compared Danshen versus an observation. Additionally, there were trials each comparing Danshen alone versus sodium alginate, Xuezhikang (XZK), or Zhibituo tablets as control groups. The results are presented in Table 3, and the complete data are presented in Figure 4.

##### LDL-Cholesterols

**Figure 5:**
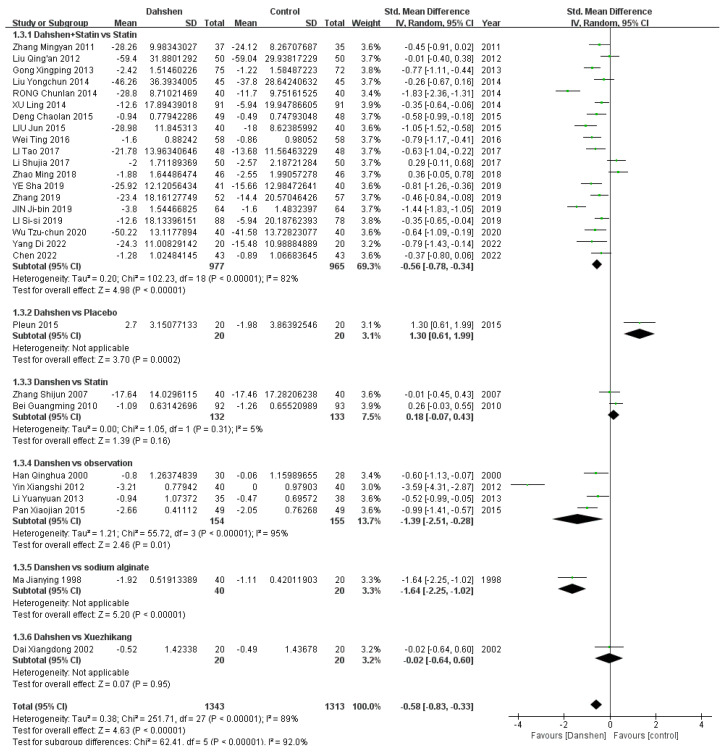
Complete data of LDL-cholesterol [10,11,12,14,15,16,17,18,19,20,21,22,23,24,25,26,27,28,29,32,33,35,36,37,38,39,40,41].

Nineteen trials compared the concurrent use of Danshen and statins versus statins alone. Two trials compared Danshen alone versus statins alone. Four trials compared Danshen alone versus the observation. Additionally, there were trials each comparing Danshen alone versus sodium alginate, Xuezhikang (XZK), or placebo as control groups. The results are presented in Table 4, and the complete data are presented in Figure 5.

##### HDL-Cholesterol

**Figure 6:**
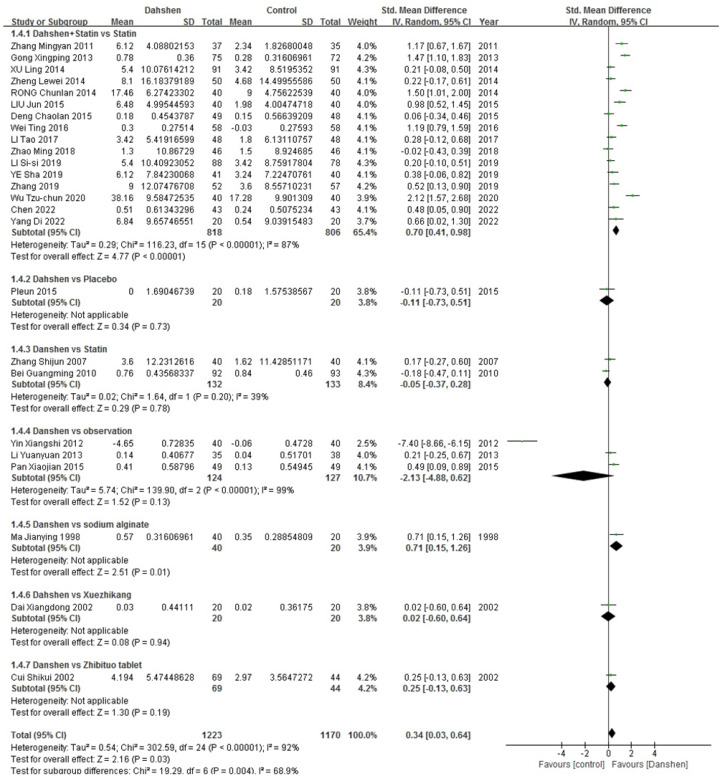
Complete data of HDL-cholesterol [10,11,12,16,17,18,21,22,23,24,25,26,27,28,29,30,32,33,35,37,38,39,40,41,42].

Sixteen trials compared the concurrent use of Danshen and statins versus statins alone. Two trials compared Danshen alone versus statins alone. Three trials compared Danshen alone versus the observation. Additionally, there were trails each comparing Danshen alone versus sodium alginate, Xuezhikang (XZK), Zhibituo tablet or placebo as control groups. The results are presented in Table 5, and the complete data are presented in Figure 6.

### 2.2. Results of In Silico Network Construction and Analysis

#### 2.2.1. ADME of Active Compounds of *Salvia miltiorrhiza*

Among the various compounds identified from *Salvia miltiorrhiza*, five key constituents—Tetrahydrotanshinone, Cryptotanshinone, Dihydrotanshinone I, Tanshinone IIa, and Tanshinone I—were selected for evaluation based on their pharmacokinetic and ADME (absorption, distribution, metabolism, and excretion) properties and HPLC fingerprints results of *Salvia miltiorrhiza* [43]. The molecular weights of these compounds ranged from 276.3 to 296.39, with Tanshinone I having the lowest molecular weight (276.3) and Cryptotanshinone the highest (296.39). Oral bioavailability (OB) values varied between 29.27% (Tanshinone I) and 52.34% (Cryptotanshinone), indicating moderate to high bioavailability across the selected constituents. The drug likeness (DL) scores ranged from 0.36 to 0.4, suggesting favorable drug-like properties. Notably, all five compounds complied with Lipinski’s rule, showing no violations, which underscores their potential as orally active therapeutic agents (Table 6).

The ADME properties of these selected Danshen constituents were also comprehensively analyzed. The absorption potential, as indicated by the Caco-2 permeability values, ranged from 0.95 to 1.05, with Tanshinone IIa and Tanshinone I exhibiting the highest permeability. Plasma protein binding (PPB) values were relatively high across all compounds, ranging from 82.424% to 89.131%, suggesting an extensive distribution. Blood–brain barrier (BBB) penetration varied, with Tanshinone IIa showing the highest potential (0.7) and Tetrahydrotanshinone the lowest (0.39) (Table 6).

Metabolically, all compounds were predicted to inhibit key cytochrome P450 enzymes (CYP1A2, CYP2C19, CYP2C9, and CYP3A4), with the exception of Tanshinone I, which did not inhibit CYP2C9, and Tetrahydrotanshinone and Cryptotanshinone, which did not inhibit CYP2D6. The half-life (T 1/2) of the compounds ranged from 1.729 h (Tetrahydrotanshinone) to 2.145 h (Tanshinone I), indicating moderate elimination rates. In terms of toxicity, human hepatotoxicity values were within acceptable ranges, from 0.73 to 0.83, and the LD50 values, representing acute toxicity, ranged from 2.525 to 2.712, indicating a relatively low toxicity profile (Table 6).

Collectively, these results highlight the favorable pharmacokinetic and safety profiles of the selected Danshen compounds, reinforcing their potential as candidates for further drug development and clinical evaluation.

#### 2.2.2. Identifying Overlapping Genes of Compounds and Dyslipidemia

**Figure 7:**
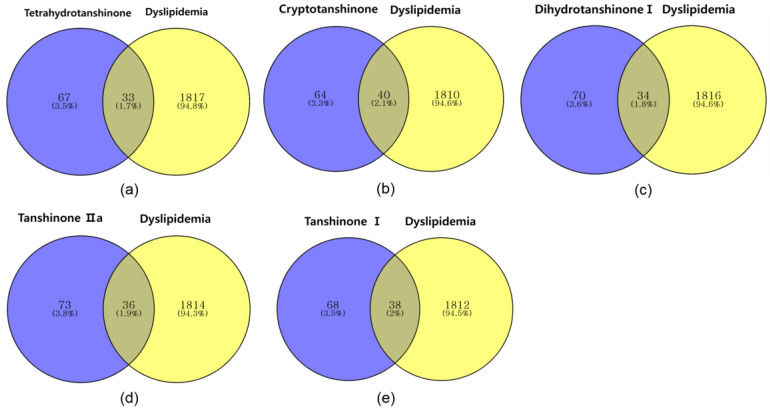
Overlapping genes between *Salvia miltiorrhiza* compounds and dyslipidemia. (**a**) Venn diagram of Tetrahydrotanshinone and dyslipidemia-related genes; (**b**) Venn diagram of Cryptotanshinone and dyslipidemia-related genes; (**c**) Venn diagram of Dihydrotanshinone I and dyslipidemia-related genes; (**d**) Venn diagram of Tanshinone IIa and dyslipidemia-related genes; (**e**) Venn diagram of Tanshinone I and dyslipidemia-related genes.

A Venn diagram analysis was conducted to identify the overlapping genes between five key compounds from Danshen (Tetrahydrotanshinone, Cryptotanshinone, Dihydrotanshinone I, Tanshinone IIa, and Tanshinone I) and dyslipidemia-related genes. The results demonstrated varying degrees of overlap, indicating the potential relevance of these compounds in modulating dyslipidemia pathways. Specifically, Tetrahydrotanshinone showed 33 overlapping genes (1.7%), Cryptotanshinone had 40 overlapping genes (2.1%), and Dihydrotanshinone I presented 34 overlapping genes (1.8%) with dyslipidemia. Similarly, Tanshinone IIa and Tanshinone I exhibited 36 (1.9%) and 38 (2.0%) overlapping genes, respectively (Figure 7).

#### 2.2.3. Network Description of Compounds and Dyslipidemia

**Figure 8:**
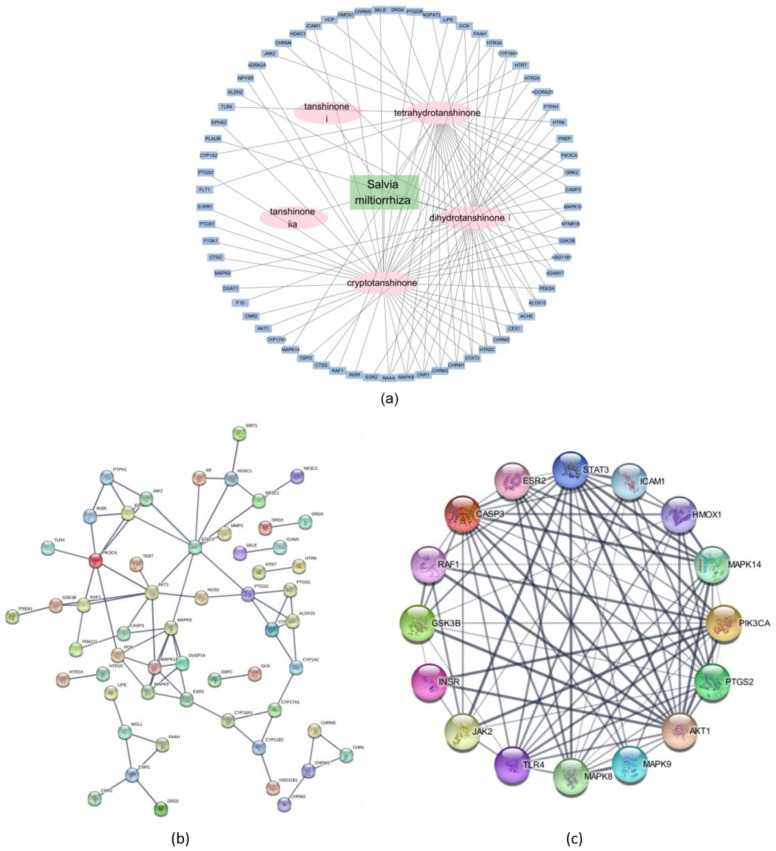
Network analysis of *Salvia miltiorrhiza* constituents and their interaction with dyslipidemia-related genes. (**a**) Interaction network of *Salvia miltiorrhiza* compounds and target genes; (**b**) protein–protein interaction network of target genes; (**c**) refined network of top 16 genes.

A network analysis was conducted to investigate the interaction between the key constituents of Danshen and dyslipidemia-associated genes. The first visualization, generated using Cytoscape, illustrates the interaction network between the five selected compounds—Tetrahydrotanshinone, Cryptotanshinone, Dihydrotanshinone I, Tanshinone IIa, and Tanshinone I—and their target genes. This network reveals the complex interconnections between the compounds and multiple dyslipidemia-related targets, underscoring the multi-target therapeutic potential of these compounds (Figure 8a).

Subsequently, a protein–protein interaction (PPI) network was constructed using the STRING database, encompassing the broader network of interactions among the identified target proteins. This PPI network highlights the interconnected nature of the dyslipidemia-related pathways and the central role of several key proteins, suggesting their importance in mediating the therapeutic effects of Danshen constituents (Figure 8b).

To further refine the analysis, we focused on the top 16 genes with the highest degree of connectivity (degree ≥ 4) within the PPI network. This refined network demonstrates the core targets that may play crucial roles in the therapeutic modulation of dyslipidemia. Key genes such as phosphatidylinositol-4,5-bisphosphate 3-kinase catalytic subunit alpha (PIK3CA), Akt Serine/Threonine Kinase 1 (AKT1), and signal transducer and activator of transcription 3 (STAT3) were identified as central nodes, suggesting their potential as critical mediators of the observed pharmacological effects. These findings provide valuable insights into the molecular mechanisms through which Danshen compounds exert their effects on dyslipidemia, offering a foundation for future experimental validation and drug development efforts (Figure 8c).

#### 2.2.4. GO and KEGG Analyses

**Figure 9:**
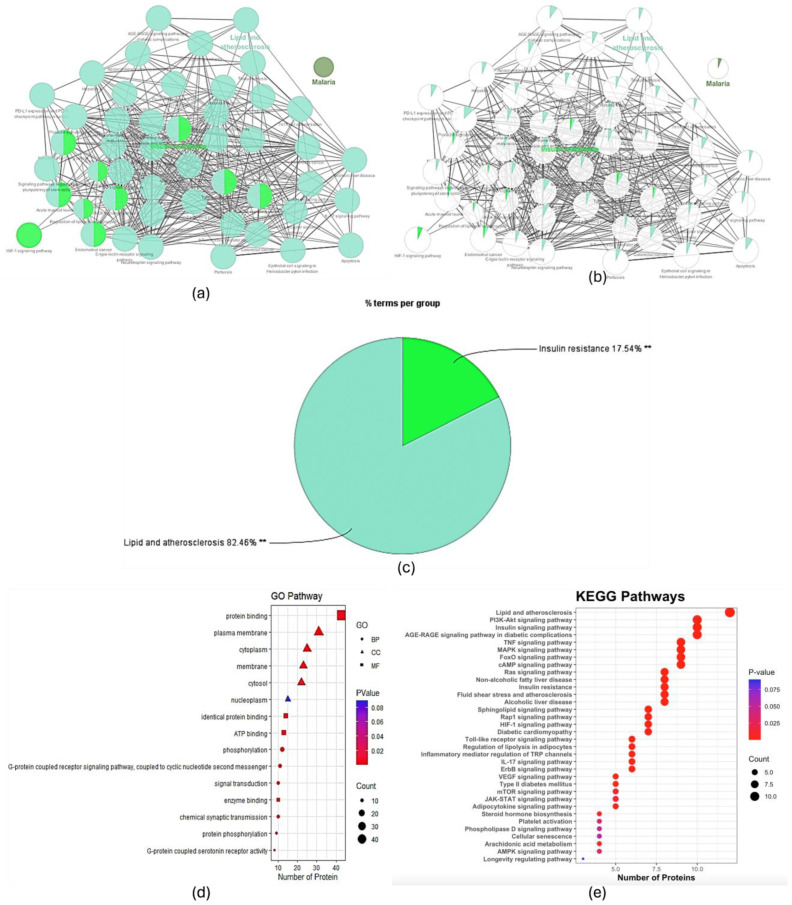
ClueGO, GO, and KEGG pathway enrichment analysis of Danshen compounds’ targets. (**a**) ClueGO network visualization of selected pathways; (**b**) proportional representation of pathways in the network; (**c**) KEGG pathway distribution with filtered terms; (**d**) GO top 5 BP, CC, and MF pathways; (**e**) KEGG analysis identifying key pathways. ** *p*-value<0.001.

A ClueGO analysis was performed to elucidate the biological pathways associated with the targets of Danshen compounds. The analysis revealed that the most enriched pathways were related to lipid and atherosclerosis (82.46%) and insulin resistance (17.54%), indicating that these pathways are significantly involved in the pharmacological effects of the compounds. The network visualization demonstrated a highly interconnected structure among the identified pathways, with lipid metabolism and insulin signaling being central nodes (Figure 9a–c).

Gene Ontology (GO) and Kyoto Encyclopedia of Genes and Genomes (KEGG) pathway enrichment analyses were conducted to explore the functional implications of the Danshen compounds’ targets. GO analysis revealed that the most enriched molecular functions were protein binding, adenosine 5′-triphosphate (ATP) binding, and enzyme binding, while the top cellular components included the plasma membrane, cytoplasm, and nucleoplasm. In terms of biological processes, signal transduction and phosphorylation were prominently enriched. The KEGG pathway analysis identified significant enrichment in pathways related to lipid and atherosclerosis, insulin resistance, and Phosphoinositide 3-kinase (PI3K)-Akt signaling (Figure 9d,e).

#### 2.2.5. Molecular Docking

**Figure 10:**
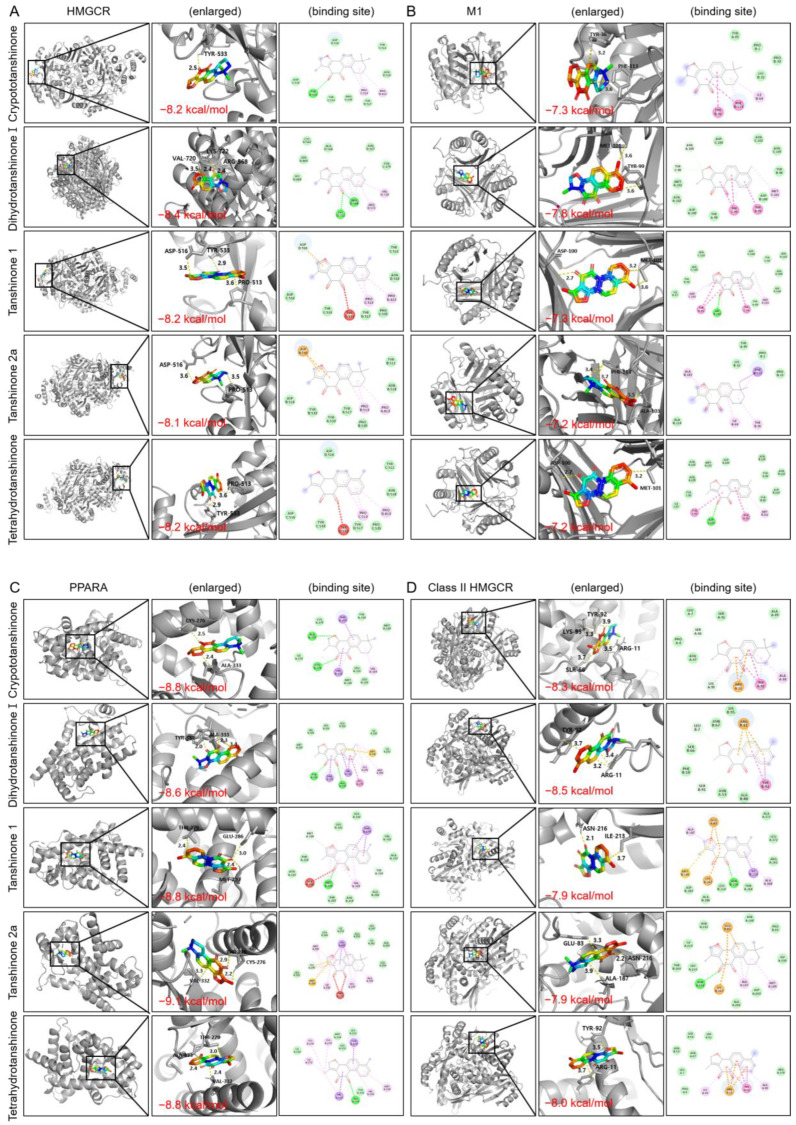

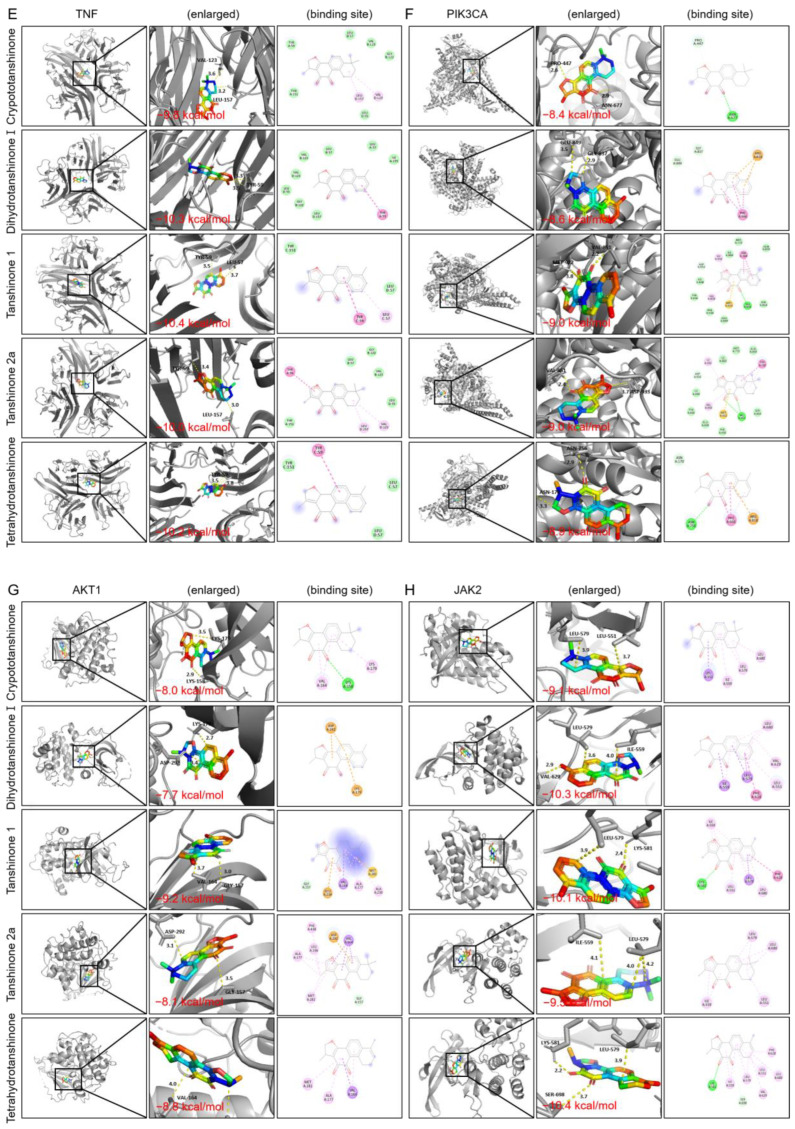

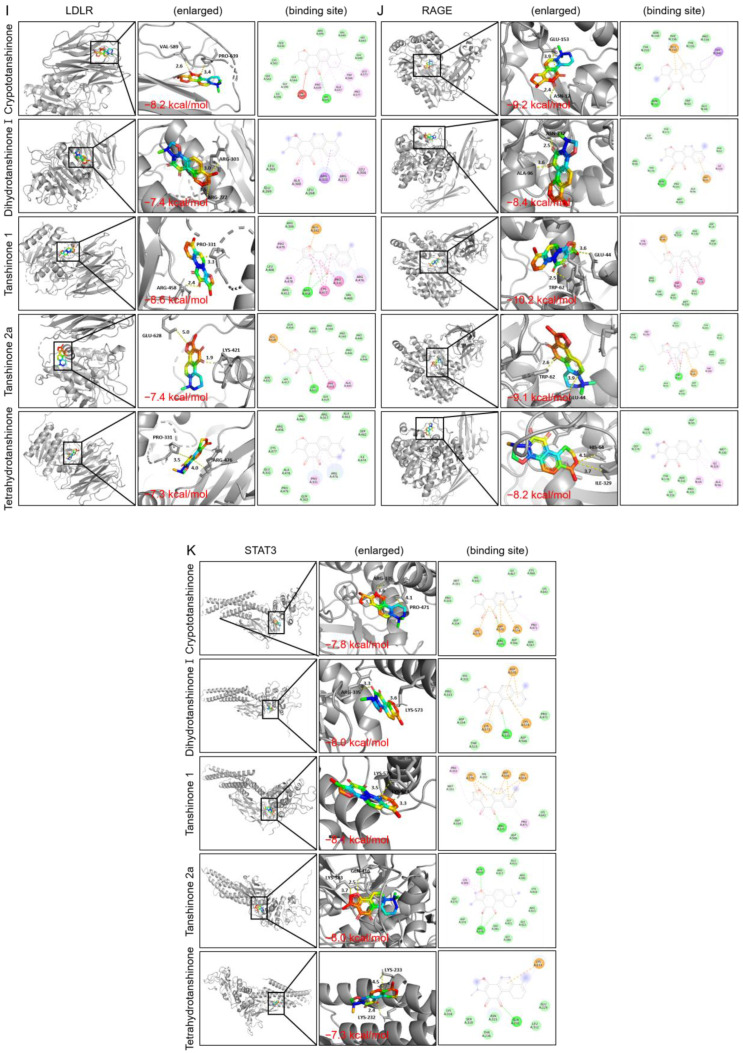
Molecular docking analysis of Danshen compounds with dyslipidemia-related protein targets. (**A**) Compound docking with HMGCR; (**B**) compound docking with class II HMGCR; (**C**) compound docking with LDLR; (**D**) compound docking with PPARA; (**E**) compound docking with JAK2; (**F**) compound docking with RAGE; (**G**) compound docking with STAT3; (**H**) compound docking with macrophage; (**I**) compound docking with TNF; (**J**) compound docking with PIK3CA; (**K**) compound docking with AKT1.

The molecular docking analysis revealed that the five Danshen compounds—Cryptotanshinone, Dihydrotanshinone I, Tanshinone I, Tanshinone IIa, and Tetrahydrotanshinone—exhibited strong binding affinities across a panel of lipid and atherosclerosis-related protein targets, which are class I HMGCR, class II HMGCR, low-density lipoprotein receptor (LDLR), peroxisome proliferator-activated receptor-α (PPARA), janus kinase2 (JAK2), and receptor for advanced glycation end products (RAGE), with binding energy values consistently lower than −7.4 kcal/mol. Binding affinities with STAT3, macrophage and tumor necrosis factor (TNF), which are insulin resistance-related proteins, demonstrated significant interactions. Proteins related to PI3K-Akt pathways also exhibited intimate interactions, which were lower than −7.7 kcal/mol. Molecular docking targets included key proteins of the PPI network including JAK2, STAT3, PIK3CA, and AKT1, suggesting their potential as effective modulators of these targets (Table 7 and Figure 10).

## 3. Discussion

Dyslipidemia is an eminent risk factor for ASCVD [44], and as it is a leading cause of mortality globally, the management of dyslipidemia is critical. Statin, an HMGCR inhibitor, is a cornerstone for treating dyslipidemia, and other lipid-lowering drugs include ezetimibe, proprotein convertase subtilisin/kexin type 9 (PCSK9) inhibitors, and bile acid sequestrants. While statins are gold standard therapy for dyslipidemia, there are several statin-associated side effects [44]. Most common side effect is statin-associated muscle symptoms that appear as myalgia, and they are reported in 5–20% of the prescribed patients. Moreover, statin use in patients with major risk factors for diabetes showed 28% increased risk for new-onset diabetes [45]. Major risk factors include high fasting glucose, obesity, an HbA1c of over 6% and metabolic syndromes, which are common predispositions in patients with abnormal lipid profiles. While studies show achieving a lower LDL-C level leads to a reduced risk of subsequent cardiovascular events [46], statin usage shows limitation in achieving therapeutic targets. A retrospective cohort study, for example, showed that only 20% of patients with statin treatment achieved the LDL cholesterol target, which is under 70 mg/dL [47]. Thus, the development of novel lipid-lowering agents as an alternative or adjuvant therapy to statin is gaining importance.

*Salvia miltiorrhiza* Burge, also known as Danshen, has long been used for relieving blood stasis and improving blood circulation in the East Asian medicines. Through scientific research, Danshen has been widely studied for its protective properties in cardiovascular diseases and diabetes, anti-oxidative effects, inhibitory effects on apoptosis, and anti-inflammatory effects [48]. To elucidate this, Tanshinone IIa, the most actively investigated constituents of Danshen, has been reported to possess a wide range of therapeutic effects in cardiovascular diseases [8], and its anti-inflammatory effects include the downregulation of the Toll-like receptor 4 (TLR4)/NF-κB pathway and the inhibition of nitric oxide (NO), interleukin (IL)-1β, IL-6 and TNF-α [49].

While Danshen, and its preparations in pill and injection formulations, is widely used in China for the treatment of dyslipidemia and ASCVD [48] and studies have elucidated the favorable effect of Danshen usage in dyslipidemia, there has been no meta-analysis on the therapeutic efficacy of Danshen against dyslipidemia. Thus, in our study, we conducted a meta-analysis of random clinical trials of Danshen on dyslipidemia through the databases of CNKI and PubMed. Among the 150 screened records, 33 studies were included in the meta-analysis, and the majority of the included studies were statin add-on studies. Danshen treatment with statin showed significant superiority over the statin group in total cholesterol, LDL, HDL, and triglycerides. The results from the meta-analysis suggested the therapeutic efficacy of Danshen as a treatment agent for dyslipidemia.

As natural products normally contain various chemical constituents, the detection of their major bioactive constituents enables us to implement a scientific investigation of their therapeutic effects. With the results from the HPLC fingerprinting of Danshen [43], we selected major constituents with a high possibility of drug utilization among several constituents; Tetrahydrotanshinone, Cryptotanshinone, Dihydrotanshinone I, Tanshinone IIa, and Tanshinone I. These constituents appeared as promising candidate components of Danshen after verification regarding absorption, distribution, metabolism, excretion, drug-likeness, and toxicity.

To further elucidate the possible treatment targets and mechanism responsible for the efficacy of Danshen against dyslipidemia, a network pharmacological analysis was implemented. We obtained overlapping genes between Danshen compounds and dyslipidemia, and through a PPI network analysis, key target genes such as JAK2, STAT3, PI3K, AKT1, and TLR4 were acquired. The KEGG pathway analysis of overlapping genes suggested relevant pathways such as lipid and atherosclerosis, PI3K-AKT, insulin signaling, advanced glycation end product (AGE)-RAGE, TNF, and mitogen-activated protein kinase (MAPK). With overlapping genes associated with the pathways of lipids and atherosclerosis, insulin resistance, hypoxia-inducible factor-1 (HIF-1), and non-alcoholic fatty liver disease (NAFLD), an additional ClueGO analysis revealed lipid and atherosclerosis as the most relevant terms and insulin resistance as the next. Other relevant pathways included AGE-RAGE, TNF, HIF-1, and adipocytokine. Based on the PPI network analysis and pathway analysis, several therapeutic target genes that Danshen might interact with were selected, and we assessed the reliability of binding them with computational molecular docking.

First, we focused on the PI3K/Akt pathway, which plays a key role in the development of atherosclerosis in several stages, such as macrophage polarization, increased intracellular lipid storage, smooth muscle cell proliferation and dysfunction [50]. Oxidized LDL induces PI3K/Akt signaling in macrophages to form foam cells and activate MAPK and the mammalian target of rapamycin (mTOR) downstream, resulting in the proliferation of arterial wall and plaque formation. PI3K/Akt plays a crucial role in regulating macrophage polarization in vascular inflammation as well through NF-κB [51]. Plaque rupture, owing to inflammation, and the following vessel obstruction and thrombosis are related to atherosclerotic progression. Restraining macrophage infiltration in plaque and promoting the autophagy of macrophages enables plaque stability, and PI3K/Akt through mTOR and TLR4 is reported to be a relevant pathway. Also, the inhibition of PI3K/Akt signaling suppresses B-cell lymphoma-extra large (Bcl-xL) and Bcl-2 expressions, which are anti-apoptotic molecules, and thus result in an increase in the apoptosis of foam cells [50,52]. Thus, PI3K/Akt-targeted agents are deemed a novel mechanism of action for treatment for atherosclerosis. All five major constituents of Danshen showed a high binding affinity with PI3K and AKT1 in the molecular docking simulation, and among them, Tanshinone I showed an especially high binding affinity of −9 and −9.2 kcal/mol, respectively.

AGE attenuates RAGE and JAK/STAT cascade expressions [53]. While the JAK/STAT pathway is involved in various processes, its role in inflammation is especially gaining attention as a key factor in inflammation. Cytokines and growth factors initiate the JAK/STAT pathway, and the downstream of JAK/STAT includes diverse genes involved in proliferation and apoptosis in atherosclerosis, such as IL-6, interferon (IFN)-γ, TNF-α and the suppressor of cytokine signaling (SOCS) [54]. The activation of RAGE and the JAK/STAT pathway gives rise to inflammation and oxidative stress, and as a result, accelerations of LDL oxidation, vascular plaque formation, smooth muscle cell proliferation and extracellular matrix production are induced [55,56,57]. Thus, regulating RAGE and JAK/STAT in atherosclerosis is deemed as an appealing therapeutic target. In our in silico studies, we observed a significantly high binding affinity of Danshen constituents with a JAK2 of over −9.1 kcal/mol. Binding powers with RAGE and STAT3 showed promising results as well.

LDL receptors in the liver plays a critical role in regulating plasma LDL levels as approximately 70% of the circulating LDL is cleared off by hepatic LDL receptors [58]. Because a small number of hepatic LDL receptors lead to elevated plasma LDL levels, the development of drugs that can increase available LDL receptors such as the PCSK9 inhibitor is one of the strategies for dyslipidemia treatment. Thus, our in silico investigation, we performed molecular docking with LDLR and showed a binding power of −7.3~−8.6 kcal/mol.

HMGCR converts HMG-CoA to mevalonic acid, and the mevalonate pathway is responsible for cholesterol biosynthesis [59]. Statin acts as an HMG-CoA reductase inhibitor, thus resulting in decreased intracellular cholesterol activate sterol regulatory element-binding proteins (SREBPs), and its binding to a sterol regulatory element results in an increased transcription of LDL receptor genes and thus an influx of plasma LDL cholesterols into the hepatocyte. While statin is a powerful HMG-CoA reductase inhibitor, HMG-CoA reductases are reported to have two classes. They share the same catalytic mechanism but have a significant difference in their three-dimensional structures and sensitivity to statin [60]. While class I HMG-CoA reductase is sensitive to the inhibition of statin, class II HMG-CoA reductase is reported to be considerably less sensitive. Molecular dockings with two classes of HMG-CoA reductase showed a considerably high binding power. High binding affinities with class II HMG-CoA reductase that showed −7.9 to −8.5 kcal/mol are especially noticeable in that they suggest a novel therapeutic target of Danshen that statin does not interact with. The in silico results suggested a high possibility of Danshen’s action on two classes of HMG-CoA reductase and LDL receptors, which is statin’s main mechanism of action. These results comply with the results of our meta-analysis which demonstrated the therapeutic efficacy of Danshen in lipid profiles when used as an adjuvant to statins compared to statin therapy alone.

Inflammation plays a crucial role in the development of ASCVD as it is related to stages such as the infiltration of monocytes, foam cell formation, and plaque vulnerability. The importance of inflammation regulation in dyslipidemia is undebatable. TNF-α activates NF-κB, and NF-κB promotes adhesion molecules such as selectin E (SELE) and intercellular adhesion molecule (ICAM)-1 to recruit monocytes into the injured sites in the endothelial cells [61]. Monocyte-derived macrophages and tissue-resident macrophages develop two phenotypes, which are M1 and M2 macrophages, and they have pro-inflammatory and anti-inflammatory characteristics, respectively [50]. Inflammatory changes in ASCVD induce an M1-dominant polarization of macrophages, which leads to plaque pathogenesis [62]. Thus, we assessed the binding affinity of major compounds of Danshen with classical macrophage M1 and major cytokine TNF for the verification of its anti-inflammatory effects, and they showed favorable results.

PPAR-α activates fatty acid beta-oxidation [63]. Due to its triglyceride-lowering effects, fibrate, which is a PPAR-α agonist, has long been utilized for dyslipidemia treatment. PPAR-α suppresses endothelin-1 expression, resulting in an alleviated smooth muscle cell proliferation. Moreover, PPAR-α has anti-inflammatory effects as it inhibits the cytokine-initiated expression of vascular adhesion molecule-1 (VCAM-1), reduces IL-6 production, and suppresses cyclooxygenase (COX)-2 activity by regulating NF-κB. Due to these beneficial effects on metabolic disorders, the PPAR-α agonist is deemed a promising agent for dyslipidemia as well. The binding affinity of Danshen constituents with PPAR-α was remarkably high, showing −8.6 to −9.1 kcal/mol.

Overall, our in silico investigation elucidated that Danshen may alleviate the development of dyslipidemia into ASCVD by suppressing macrophage polarization, apoptosis, and smooth muscle cell proliferation via modulating PI3K/Akt. Also, the modulation of Danshen on RAGE and the JAK/STAT pathway is also suggested, resulting in the inhibition of inflammation and oxidative stress and an acceleration of vascular plaque formation. Danshen may alleviate inflammation by suppressing key inflammatory cytokine TNF and classical macrophage M1. Regarding cholesterol biosynthesis, Danshen may provide an additional lipid-lowering effect as a class II HMG-CoA reductase inhibitor. Also, Danshen may facilitate the beta-oxidation of fatty acids and the elimination of serum LDL by regulating PPAR-α and LDLR. These elucidated potential therapeutic mechanisms suggest further investigational subjects in understanding the therapeutic effect of Danshen in dyslipidemia.

There are a few limitations in our investigation. Our investigation on target genes and pathways was based on computational analysis and thus warrants careful interpretation. Although network pharmacological methods are widely used for drug research, it is important to recognize clear limitations of these techniques, such as data restrictions and data discrepancies [64]. Also, our investigation of Danshen focused on several potential active components of *Salvia miltiorrhiza*, while *Salvia miltiorrhiza* as whole includes various constituents. As many traditional medicines act through multiple bioactives and targets, an investigation on several selected bioactives may not be sufficient to explain the therapeutic effect of *Salvia miltiorrhiza* as a whole [64]. However, through a meta-analysis of randomized clinical trials, we were able to verify the therapeutic effect of Danshen against dyslipidemia to some extent. Our investigation was the first meta-analysis that assessed the therapeutic efficacy of Danshen on dyslipidemia to the best of our knowledge. In addition, we suggested possible therapeutic components, key targets and mechanisms and pathways of Danshen for the treatment of dyslipidemia. We hope our investigation will contribute to furthering the understanding of the mechanism of action of Danshen, and future in vitro or in vivo studies for the verification of the suggested therapeutic targets and pathways of Danshen are warranted.

## 4. Materials and Methods

### 4.1. Research Workflow Through Integrated Methodology

This study utilized both in silico modeling and a meta-analysis to assess the effect of *Salvia miltiorrhiza* on dyslipidemia, the latter following these steps: The first step involved identifying randomized controlled trials (RCTs) that explored the effect of *Salvia miltiorrhiza* on dyslipidemia. This initial phase ensured that there was a sufficient pool of data for a thorough analysis. Using the Cochrane’s Review Manager program (The Cochrane Collaboration, England and Wales), data from the selected trials were then systematically reviewed and analyzed, focusing on various lipid parameters to determine the effects of *Salvia miltiorrhiza*. Lastly, the study used in silico trials to predict and further investigate the estimated effects of *Salvia miltiorrhiza* for dyslipidemia.

### 4.2. Data Sources and Search Strategy

The systematic review of clinical trials was conducted following the PRISMA 2020 guidelines for systematic reviews and meta-analyses. All researchers actively participated in the data collection. Studies assessing the efficacy and safety of *Salvia miltiorrhiza* for dyslipidemia were identified using two databases, PubMed and the Chinese National Knowledge Infrastructure (CNKI), covering publications up to May 2024.

### 4.3. Study Selection

#### 4.3.1. Type of Studies

In this study, RCTs evaluating the efficacy and safety of *Salvia miltiorrhiza* for dyslipidemia were only included, with no restrictions on language or publication date. Studies were excluded if they met any of the following criteria: (a) were not randomized control trials; (b) unrelated to dyslipidemia; (c) did not use *Salvia miltiorrhiza* as the primary intervention; (d) involved non-oral administration methods; (e) were case reports or reviews; (f) involved animal studies; (g) did not consider lipid profiles as primary outcomes; and (h) were not published in scientific peer-reviewed journals, including theses and dissertations.

#### 4.3.2. Type of Participants

The review included studies without restrictions based on age, gender, or race but only those with patients diagnosed with dyslipidemia using validated criteria. Studies involving participants with diabetes or acute pancreatitis were excluded in order to maintain focus on dyslipidemia.

#### 4.3.3. Type of Interventions

RCTs that investigated the effects of *Salvia miltiorrhiza* as the main treatment in comparison to a control group were included. Various forms of the intervention, such as decoctions, granules, capsules, and tablets, were accepted. There were no limitations on dosage or treatment duration, but only oral forms of administration were considered. Thus, trials involving injections of *Salvia miltiorrhiza* were excluded. Similarly, trials that combined the use of other therapies, such as non-drug treatments, acupuncture, massage, or additional herbal ingredients, were not included.

#### 4.3.4. Type of Outcome Measures

The primary outcomes analyzed were lipid profiles, specifically total cholesterol (TC), triglycerides (TGs), high-density lipoprotein cholesterol (HDL-C), and low-density lipoprotein cholesterol (LDL-C).

### 4.4. Methodological Quality Assessment

The methodological quality of each included study was independently assessed by all researchers using the updated version of the Risk of Bias tool (RoB 2.0) for randomized trials. RoB 2.0 focuses on five potential sources of bias: (a) biases related to the randomization process, (b) biases arising from deviations in allocation concealment, (c) biases due to insufficient blinding, (d) biases from incomplete outcome data, and (e) biases in selective outcome reporting. Studies were classified into three levels of methodological quality—“high risk of bias,” “low risk of bias,” and “uncertain risk of bias”—using color codes of red, green, and yellow, respectively. Disagreements among researchers were resolved through consensus, guided by the corresponding author.

### 4.5. Quality of Evidence According to Outcome Measures

The quality of evidence for each outcome was assessed using the GRADE (Grading of Recommendations Assessment, Development, and Evaluation) system. GRADE categorizes evidence into four levels: very low, low, moderate, and high. The level of evidence can be downgraded based on factors like potential bias, an inconsistency of the results, the lack of direct evidence, imprecision, or publication bias.

### 4.6. Statistical Analysis

Statistical analyses were performed using the RevMan software (ver. 5.4). Trials were categorized based on the type of intervention and control group. The relative risk (RR) and 95% confidence intervals (CIs) were calculated for lipid parameters, including total cholesterol (TC), triglycerides (TGs), HDL-C, and LDL-C. To combine the results measured with different units, the standardized mean difference (SMD) and 95% confidence intervals (CIs) were computed. The analysis detected heterogeneity among the primary outcomes across the randomized controlled trials, and a contour-enhanced funnel plot was created for further analysis.

### 4.7. Network Pharmacology Analysis of Anti-Dyslipidemia Mechanisms

A network pharmacology analysis was conducted to investigate how Danshen may treat dyslipidemia. The key chemical constituents in Danshen were sourced from the TCMSP (Traditional Chinese Medicine Systems Pharmacology database and analysis platform, https://tcmsp-e.com/, accessed on 23 August 2024), and results from the HPLC fingerprinting of Danshen [43]. In this study, the compounds of Danshen were screened based on the following criteria obtained from the TCMSP database, oral bioavailability (OB) ≥ 30%, Caco-2 permeability ≥ −0.4, and drug likeness (DL) ≥ 0.18, and then only the compounds that also exhibited peaks in the HPLC analysis of Danshen were selected. Additionally, tanshinone I, a known marker compound of Danshen, was included for further analysis. Absorption, distribution, metabolism, and excretion (ADME) predictions for these five compounds were evaluated using the SwissADME platform (http://www.swissadme.ch/, accessed on 23 August 2024). In the SwissADME platform, compounds were evaluated according to the ADME rules of Lipsinski. The SwissTargetPrediction platform (http://www.swisstargetprediction.ch, accessed on 23 August 2024.) (in the “Homo sapiens” mode) was used to predict potential target genes, and the target information was standardized using the Uniprot database (http://www.uniprot.org, accessed on 23 August 2024). Dyslipidemia-related target genes were retrieved from the GeneCards database (http://www.genecards.org, accessed on 23 August 2024) using “dyslipidemia” as the search term.

Using the “Bioinformatics for Genomics and Proteomics site” (https://bioinfogp. cnb.csic.es/tools/venny/, accessed on 23 August 2024), Venn diagrams showing the overlap between key constituents and dyslipidemia were generated. A network illustrating the interrelation between chemicals and targets was constructed using Cytoscape (version 3.10.2; https://cytoscape.org/, accessed on 23 August 2024) to visualize the relationships between the core constituents of Danshen and dyslipidemia targets. The STRING database (12.0; https://string-db.org/, accessed on 23 August 2024) was used to analyze the interaction gene targets of the major constituents and dyslipidemia, filtering by “Homo sapiens”. Disconnected nodes in the network were set to “hide”, and the confidence score was set to ≥ 0.7. The protein–protein interaction (PPI) network was constructed, redundant nodes were removed, and the top 16 genes with the highest connectivity (degree ≥ 4) were analyzed in Cytoscape.

To characterize gene targets by function, including biological processes, cellular components, and molecular functions, we used Gene Ontology (GO) functional analysis. A graph was created by selecting the top 4 counts from each category. Kyoto Encyclopedia of Genes and Genomes (KEGG) enrichment analysis was employed to identify shared targets between the core constituents and dyslipidemia within signaling pathways. The gene lists for four pathways—lipid and atherosclerosis, insulin resistance, HIF-1 signaling pathway, and NAFLD—strongly linked to dyslipidemia were analyzed using ClueGO in Cytoscape, with a data selection threshold of *p* < 0.05.

### 4.8. Docking Interactions of Danshen Compounds and Target Proteins

Based on the results of the KEGG analysis, we examined the detailed mechanisms of the lipid and atherosclerosis pathway, which showed the highest count, using a pathway map (https://www.genome.jp/pathway/map05417, accessed on 23 August 2024). Several key genes involved in the core processes were selected, and molecular docking was performed with the main constituents of Danshen. The three-dimensional structures of Danshen compounds—Cryptotanshinone, Dihydrotanshinone I, Tanshinone I, Tanshinone IIa, and Tetrahydrotanshinone—were downloaded from the PubChem database (http://pubchem.ncbi.nlm.nih.gov/, accessed on 23 August 2024), while the 3D structures of proteins, including HMGCR (PDB ID: 1DQA and 1T02), LDLR (PDB ID: 3P5B), PPARA (PDB ID: 1I7G), JAK2 (PDB ID: 4FVQ), RAGE (PDB ID: 3O3U), STAT3 (PDB ID: 1BG1), macrophage (PDB ID: 1GD0), TNF (PDB ID: 2AZ5), PIK3CA (PDB ID: 8TSA), and AKT1 (PDB ID: 4GV1), were provided by the PDB database (http://rcsb.org), accessed on 8 August 2024. The criteria for selecting PDB entries for each protein are as follows: First, proteins are searched in the UniProt database (https://www.uniprot.org/, accessed on 23 August 2024), ensuring that the organism is specified as Homo sapiens (Human). From the provided PDB list, only entries that utilize the X-ray diffraction method and have a resolution of 3.0 Å or lower are considered. Subsequently, the RCSB PDB links for the qualifying PDB files are accessed to download them in PDB format. Prior to molecular docking, unnecessary protein domains were removed, and hydrogenation was performed using the Biovia Discovery Studio Visualizer. Molecular docking was conducted using PyRx to assess binding affinity. The interaction sites of the ligand and corresponding distances were analyzed using the receptor–ligand interactions tab in Biovia Discovery Studio Visualizer, where a 2D diagram was created. Additionally, the simulated binding interactions were visualized in PyMOL.

## 5. Conclusions

This study suggests that Danshen has potential as an adjunct therapy for treating dyslipidemia, particularly when combined with statins. The meta-analysis showed that Danshen significantly improves lipid profiles, including reductions in total cholesterol, LDL-C, and triglycerides while increasing HDL-C, compared to statins alone. Mechanistic analyses suggest that Danshen influences key pathways involved in lipid metabolism, inflammation, and atherosclerosis, such as PI3K/Akt, JAK/STAT, and HMGCR pathways. These findings indicate that Danshen may offer additional therapeutic benefits beyond conventional statin treatment, potentially improving cardiovascular outcomes for patients with dyslipidemia. Larger clinical trials with longer follow-up periods are also necessary to assess Danshen’s long-term impact on cardiovascular outcomes.

## Figures and Tables

**Table 1:** Characteristics of each experiment.

First Author	Participants	Number of Cases/Age, Years			Intervention Methods		Observation Period	Outcome Indicators
		Trial Group	Control Group		Trial Group	Control Group		
Chen, 2022 [10]	Patients aged 51–83 years	63.97 ± 5.48	64.38 ± 5.79		Standard treatment (anticoagulants, antiplatelet aggregation agents, blood sugar-lowering agents, antihypertensive drugs) with Danshen dripping tablets 10 T tid	Standard treatment (anticoagulants, antiplatelet aggregation agents, blood sugar-lowering agents, antihypertensive drugs) with Atorvastatin 20 mg qd	12 weeks	Blood lipid levels via TC, TG, HDL-C, LDL-C
Deng Chaolan, 2015 [11]	Patients aged 41–80 years	49/61.58 ± 7.30	48/61.16 ± 7.15		Compound Danshen soft capsules 27 mg tid	Atorvastatin 10 mg qd	12 weeks	Blood lipid levels via TC, TG, HDL-C, LDL-C
Gong Xingping, 2013 [12]	Patients aged 34–76 years	77/ 45.7 ± 8.2	73/ 46.2 ± 8.6		Simvastatin 20 mg qd with compound Danshen dripping tablet 10 T tid	Simvastatin 20 mg qd	12 weeks	Blood lipid levels via TC, TG, HDL-C, LDL-C
Guo Hongmei, 2016 [13]	76 patients	38/57.4 ± 5.2	38/56.1 ± 4.8		Simvastatin 10 mg qd with compound Danshen dripping tablets 10 T tid	Simvastatin 10 mg qd	12 weeks	Blood lipid levels via TC, TG,
Jin Ji-bin, 2019 [14]	Patients aged 30–65 years	64/45.8 ± 8.5	64/46.7 ± 7.9		Simvastatin 5 mg qd with compound Danshen dripping tablets 10 T tid	Simvastatin 5 mg qd	12 weeks	Blood lipid levels via TG, TC, LDL-C
Li Shujia, 2017 [15]	Patients aged 45–85 years	50/62.5 ± 5.1	50/61.9 ± 3.6		Rosuvastatin 10 mg/20 mg/40 mg qd with compound Danshen dripping tablets 10 T tid	Rosuvastatin 10 mg/20 mg/40 mg qd	8 weeks	Blood lipid levels via TG, TC, LDL-C
Li Si-si, 2019 [16]	Patients average age of 56.4 ± 7.9 years	not mentioned	not mentioned		Baseline medications (enteric-coated aspirin 100 mg qd and isosorbide mononitrate 40 mg qd) with compound Danshen dripping tablets 270 mg tid	Baseline medications (enteric-coated aspirin 100 mg qd and isosorbide mononitrate 40 mg qd) with Rosuvastatin 10 mg qd	24 weeks	Blood lipid levels via TG, LDL, HDL, TC
Li Tao, 2017 [17]	Patients aged 42–81 years	48/59.2 ± 6.1	48/60.4 ± 5.4		Capsules containing compound Danshen dripping capsules 10 C tid	Simvastatin 20 mg/day	12 weeks	Blood lipid levels via TC, TG, HDL-C, LDL-C
Liu Jun, 2015 [18]	80 patients	40/54.8 ± 11.6	40/53.8 ± 9.4		Simvastatin 20 mg qd with compound Danshen dripping pill 10 C tid	Simvastatin 20 mg qd	8 weeks	Blood lipid levels via TG, TC, LDL-C
Liu Qingan, 2012 [19]	102 patients	52/average age 51.7	50/average age 59.8		Simvastatin 10 mg qd with complex Danshen preparation 25 mg tid	Simvastatin 10 mg qd	20 weeks	Blood lipid levels via TG, LDL-C
Liu Yongchun, 2014 [20]	90 patients	45/65.4 ± 5.1	45/64.8 ± 3.7		Rosuvastatin calcium 10 mg qd with compound Danshen dripping pill 270 mg tid	Rosuvastatin 10 mg qd	12 weeks	Blood lipid levels via TG, TC, LDL-C
Rong Chunlan, 2014 [21]	Patients aged 45–77 years	40/57.33 ± 3.29	40/57.33 ± 3.29		Standard treatment with compound Danshen dripping capsule 10 C tid	Standard treatment (β-blockers, calcium channel blockers, antiplatelet agents, statins)	4 weeks	Blood lipid levels via TC, TG, HDL-C, LDL-C
Wei Ting, 2016 [22]	Patients aged 45–86 years	58/62.8 ± 11.5	58/64.7 ± 12.8		Simvastatin 10 mg qd with compound Danshen dripping capsules 10 C tid	Simvastatin 10 mg qd	12 weeks	Blood lipid levels via TG, TC, LDL-C
Wu Zichun, 2020 [23]	Patients aged 52–81 years	65.42 ± 2.44	66.42 ± 3.21 years		Conventional treatment with Danshen dripping capsules 10 C tid	Conventional treatment with Rosuvastatin Calcium 10 mg qd	8 weeks	Blood lipid levels via TC, TG, HDL-C, LDL-C
Xu Ling, 2014 [24]	182 patients	91 (randomized)	91 (randomized)		Rosuvastatin calcium 10 mg qd with compound Danshen dripping capsule 10 C tid	Rosuvastatin 10 mg qd	8 weeks	Blood lipid levels via TC, TG, HDL-C, LDL-C
Yang Di 2022 [25]	Patients aged 36–82 years	63. 18 ± 4. 10	62. 38 ± 4. 28		Danshen dripping tablets 270 mg tid	Atorvastatin 20 mg qd	12 weeks	Blood lipid levels via TC, TG, HDL-C, LDL-C
Ye Sha, 2019 [26]	Patients aged <75 years	41/59.2 ± 6.1	40/60.4 ± 5.4		Compound Danshen dripping capsules 10 C tid	Trimetazidine 20 mg tid	12 weeks	Blood lipid levels via TC, TG, HDL-C, LDL-C
Zhang, 2019 [27]	Patients aged 52–89 years	65.8 ± 9.7	67.5 ± 7.6		Danshen dripping pills 27 mg bid	Atorvastatin 10 mg qd	12 weeks	Blood lipid levels via TG, TC, LDL-C
Zhang Mingyan, 2011 [28]	Patients aged 31–69	37/56 ± 11.45	35/55 ± 10.78		Lovastatin 20 mg qd with compound *Salvia miltiorrhiza* dripping tablets 10 T tid	Lovastatin 20 mg qd	8 weeks	Blood lipid levels via TG, TC, LDL-C, HDL-C
Zhao Ming, 2018 [29]	Patients aged 46–83 years	46/61.82 ± 4.90	46/61.95 ± 4.92		Rosuvastatin 10–40 mg qd with compound Danshen driping tablets 10 T tid	Rosuvastatin 10–40 mg qd	8 weeks	Blood lipid levels via TG, LDL-C, HDL-C
Zheng Lewei, 2014 [30]	100 patients aged 60~86(72 ± 2.6)	50 (randomized)	50 (randomized)		Atorvastatin calcium 10 mg qd with compound Danshen dripping capsules 10 C tid	Atorvastatin Calcium 20 mg qd	8 weeks	TC, TG, HDL-C
Li Xiaoyang, 2003 [31]	Patients average age under 60	20	20		Complex Salvia ginseng preparation 30 C tid	Inositol nicotinate 0.6~1.2 g tid	8 weeks	Blood lipid levels via TG, TC,
Pleun, 2015 [32]	Patients aged 40–70 years	Randomized double-blind placebo-controlled crossover study *n*= 20			Danshen capsules 500 mg (4 capsules) tid	Placebo capsules 500 mg (4 capsules) tid	4 weeks	Blood lipid levels via TG, TC, LDL-C, HDL-C
Bei Guangmin, 2010 [33]	Patients aged 42–65	92/52.9 ± 7		93/54.3 ± 6.4	Candesartan ester capsules 8 mg qd, Hydrochlorothiazide 10 mg qd, long-acting nifedipine 10 mg bid with Danshen tablets 4 T tid	Candesartan ester capsules 8 mg qd, Hydrochlorothiazide 10 mg qd, Long-acting nifedipine 10 mg bid, Atorvastatin 10 mg qd	24 weeks	Blood lipid levels via TG, TC, LDL-C, HDL-C
Zhang Huina, 2020 [34]	No information	63.74 ± 3.28		63.52 ± 3.79	Danshen dripping capsules 10 mg tid	Conventional symptomatic treatment (Betaloc (metoprolol), aspirin, lifestyle adjustment, low-sodium, low-fat diet, metformin, nifedipine, simvastatin 10 mg qd)	4 weeks	Blood lipid levels via TC, TG, HDL-C, LDL-C
Zhang Shijun, 2007 [35]	Patients aged 26–63	40/38 ± 10.2		41/39 ± 11.8	Composite Salviae dropping pills 10 C tid	Simvastatin 20 mg qd	12 weeks	Blood lipid levels via TG, TC, LDL-C, HDL-C
Han Qinghua, 2000 [36]	Patients aged 50–70	30/64.2 ± 0.3		28/63.8 ± 0.3	Compound Danshen dropping capsules 10 C tid	No treatment	4 weeks	Blood lipid levels via TG, TC, LDL-C
Li Yangyuan, 2013 [37]	73 patients	35/60.7 ± 13.6		38/59.2 ± 11.4	Atorvastatin 10 mg qd with compound Danshen dripping capsules 10 C tid	Atorvastatin 10 mg qd	4 weeks	Blood lipid levels via TG, TC, LDL-C, HDL-C
Pan Xiaojian, 2015 [38]	98 patients	49/67.3 ± 3.2		49/67.1 ± 4.2	Atorvastatin 10 mg qd with compound Danshen tablets 10 T tid	Atorvastatin 10 mg qd	20 weeks	Blood lipid levels via TG, TC, LDL-C, HDL-C
Yin Xiangshi, 2012 [39]	80 patients aged 58–75	40		40	Standard treatment (aspirin, β-blockers, calcium channel blockers, nitrates with compound Danshen preparation 10 T tid	Standard treatment (aspirin, β-blockers, calcium channel blockers, nitrates)	4 weeks	Blood lipid levels via TG, TC, LDL-C, HDL-C
Ma Jianying, 1998 [40]	Patients aged 40–45 years	40		20	Complex Salvia ginseng preparation 10 T tid	Sodium alginate double ester capsule 50 mg tid	8 weeks	Blood lipid levels via TG, TC, LDL-C, HDL-C
Dai Xiangdong, 2002 [41]	No information	20/56.3 ± 8.5		20/58.7 ± 7.5	Danshen capsules 10 C tid	Xuezhikang 0.6 g bid	4 weeks	Blood lipid levels via TG, TC, LDL-C, HDL-C
Cui Shikui, 2002 [42]	Patients aged 36–79	69/58		44/51	Compound Danshen preparation 10 T tid	Zhibitu capsule 1 C bid	4 weeks	Blood lipid levels via TG, TC, LDL-C

**Table 2:** Effective rate of total cholesterol.

	Std. Mean Difference	Heterogeneity
Danshen + Statin vs. Statin (*n* = 21)	−0.65 [−0.92, −0.38]	*p* < 0.00001, I^2^ = 89%
Danshen vs. Placebo (*n* = 2)	0.41 [−1.01, 1.84]	*p* = 0.0004, I^2^ = 92%
Danshen vs. Statin (*n* = 3)	0.08 [−0.13, 0.28]	*p* = 0.48, I^2^ = 0%
Danshen vs. Observation (*n* = 4)	−0.78 [−1.01, −0.55]	*p* = 0.95, I^2^ = 0%
Danshen vs. Sodium Alginate (*n* = 1)/ Xuezhikang (*n* = 1)/ Zhibituo Tablet (*n* = 1)		
Total	−0.55 [−0.77, −0.33]	*p* < 0.00001, I^2^ = 89%

**Table 3:** Effective rate of triglycerides.

	Std. Mean Difference	Heterogeneity
Danshen + Statin vs. Statin (*n* = 17)	−0.67 [−0.80, −0.54]	*p* = 0.02, I^2^ = 46%
Danshen vs. Placebo (*n* = 2)	−0.32 [−0.68, 0.04]	*p* = 0.97, I^2^ = 0%
Danshen vs. Statin (*n* = 3)	−1.13 [−3.06, 0.79]	*p* < 0.00001, I^2^ = 98%
Danshen vs. Observation (*n* = 4)	−1.44 [−2.53, −0.35]	*p* < 0.00001, I^2^ = 94%
Danshen vs. Sodium Alginate (*n* = 1)/ Xuezhikang (*n* = 1)/Zhibituo Tablet (*n* = 1)		
Total	−0.73 [−0.96, −0.50]	*p* < 0.00001, I^2^ = 88%

**Table 4:** Effective rate of LDL-cholesterol.

	Std. Mean Difference	Heterogeneity
Danshen + Statin vs. Statin (*n* = 19)	−0.56 [−0.78, −0.34]	*p* < 0.00001, I^2^ = 82%
Danshen vs. Statin (*n* = 2)	0.18 [−0.07, 0.43]	*p* = 0.31, I^2^ = 5%
Danshen vs. Observation (*n* = 4)	−1.39 [−2.51, −0.28]	*p* < 0.00001, I^2^ = 95%
Danshen vs. Sodium Alginate (*n* = 1)/ Xuezhikang (*n* = 1)/Placebo (*n* = 1)		
Total	−0.58 [−0.83, −0.33]	*p* < 0.00001, I^2^ = 89%

**Table 5:** Effective rate of HDL-cholesterol.

	Std. Mean Difference	Heterogeneity
Danshen + Statin vs. Statin (*n* = 16)	0.70 [0.41, 0.98]	*p* < 0.00001, I^2^ = 87%
Danshen vs. Statin (*n* = 2)	−0.05 [−0.37, 0.28]	*p* = 0.20, I^2^ = 39%
Danshen vs. Observation (*n* = 3)	−2.13 [−4.88, 0.62]	*p* < 0.00001, I^2^ = 99%
Danshen vs. Sodium Alginate (*n* = 1)/ Xuezhikang (*n* = 1)/ Zhibituo Tablet (*n* = 1)/Placebo (*n* = 1)		
Total	0.34 [0.03, 0.64]	*p* < 0.00001, I^2^ = 92%

**Table 6:** Physicochemical properties, ADME profiles, and toxicity Evaluation of selected *Salvia miltiorrhiza* constituents.

Molecular Name		Tetrahydrotanshinone	Cryptotanshinone	Dihydrotanshinone I	Tanshinone IIa	Tanshinone I
MW		280.34	296.39	278.32	294.37	276.3
OB (%)		38.75	52.34	45.04	49.89	29.27
DL		0.36	0.4	0.36	0.4	0.36
Lipinski		Yes; 0 violations	Yes; 0 violations	Yes; 0 violations	Yes; 0 violations	Yes; 0 violations
Absorption	Caco-2	0.96	0.95	0.95	1.05	1.05
Distribution	PPB	82.42	88.37	86.71	89.13	87.91
	BBB	0.39	0.51	0.43	0.7	0.53
Metabolism	CYP1A2 inhibitor	Yes	Yes	Yes	Yes	Yes
	CYP2C19 inhibitor	Yes	Yes	Yes	Yes	Yes
	CYP2C9 inhibitor	Yes	Yes	Yes	Yes	No
	CYP2D6 inhibitor	No	No	Yes	Yes	No
	CYP3A4 inhibitor	Yes	Yes	Yes	Yes	Yes
Excretion	T 1/2	1.729	1.886	1.919	2.084	2.145
Toxicity	Human hepatoxicity	0.758	0.73	0.826	0.776	0.83
	LD50	2.583	2.605	2.562	2.712	2.525

MW, molecular weight; OB, oral bioavailability; DL, drug likeness; Lipinski, Lipinski’s rule of five compliance; Caco-2, Caco-2 permeability; PPB, plasma protein binding; BBB, blood–brain barrier penetration; CYP, cytochrome P450 enzyme inhibition status, indicating potential drug–drug interactions; T 1/2, half-Life; LD50, median lethal dose.

**Table 7:** Binding affinities of Danshen compounds to dyslipidemia-related protein targets.

Target Name	PBD ID	Binding Affinity (kcal/mol)				
		Crypto- Tanshinone	Dihydro- Tanshinone I	Tanshinone I	Tanshinone IIa	Tetrahydro- Tanshinone
Lipid and atherosclerosis-related proteins						
HMGCR	1dqa	−8.2	−8.4	−8.2	−8.1	−8.2
Class II HMGCR	1t02	−8.3	−8.5	−7.9	−7.9	−8
LDLR	3p5b	−8.2	−7.4	−8.6	−7.4	−7.3
PPARA	1i7g	−8.8	−8.6	−8.8	−9.1	−8.8
JAK2	4fvq	−9.1	−10.3	−10.1	−9.5	−10.4
RAGE	3o3u	−9.2	−8.4	−10.2	−9.1	−8.2
Insulin resistance-related proteins						
STAT3	1bg1	−7.9	−8	−8.1	−8	−7.3
Macrophage	1gd0	−7.3	−7.8	−7.3	−7.2	−7.2
TNF	2az5	−9.8	−10.3	−10.4	−10	−10.2
PI3K-Akt-related proteins						
PIK3CA	8tsa	−8.4	−8.6	−9	−9	−8.9
AKT1	4gv1	−8	−7.7	−9.2	−8.1	−8.8

## Data Availability

Data are contained within the article.
